# The Treatment of Tuberculosis

**Published:** 1913-03-15

**Authors:** Walter H. Fearis

**Affiliations:** Frating


### The Editor's Letter-Box.

### "The Treatment of Tuberculosis."

*To the Editor of* The Hospital.

Sir,—With reference to the review of my book, "The Treatment of Tuberculosis by Means of the Immune Substances (I.K.) Therapy," which appeared in your issue of February 15, there are some serious inaccuracies which, owing to the scientific importance of the subject, call for comment on my part.

Although your reviewer is unable to produce any evidence from my book which warrants such a conclusion, he writes of my unacquaintance with modern medical research. For the past three and a half years I have, under Spengler's guidance, devoted myself exclusively to research work on immunity, especially with reference to tuberculosis, so I ought to know a little about the subject on which I have worked and written.

With reference to Spengler's researches on the rôle played by the erythrocytes in the generation and accumulation of the tuberculosis immune substances, your reviewer suggests that Spengler has been misled by the erythrocythæmia which is an effect of high altitude. If he had grasped the nature of Spengler's researches he would have seen that, from the point of view of the latter, erythrocythæmia is of no importance whatever. The number of erythrocytes in a blood specimen has nothing to do with these investigations: it is the relative amounts of immune substances present in the erythrocytes and serum respectively of a specimen of blood which is all important. Further, Spengler's theory is based mainly on investigations of the daily variations in the relative amounts of immune substances present in the erythrocytes and serum respectively, which are determined by serial daily observations of one and the same person's blood, as I clearly explained on pp. 40-41 of my book. The fact that the erythrocytes often contain one million times more tuberculosis immune substances than the corresponding serum has been confirmed by Fuchs-Wolfring's extensive researches, some of which were carried out at places of low altitude—*e.g.* Paris. Investigations which have been made by me in England and in Davos have yielded similar results. In Professor Castaigne's laboratory, Paris, further confirmatory results are being obtained.

Your reviewer writes: "Do all workers, or even most workers, use in consumption P.T.O. instead of human tuberculin? By no means." In my book (p. 7) I have stated that bovine tuberculins are considered the best for *most*—but not for *all*—cases of tuberculosis. Of the weighty evidence supporting my statement I need, perhaps, only mention the fact that Dr. Camac Wilkinson and his numerous followers, who have done such extensive and excellent work with tuberculin, commence the treatment of most of their cases with bovine tuberculin. But I have not stated that bovine tuberculin alone should be used. In Chapter V. I have emphasised the importance of Spengler's work, showing that the best results with tuberculin treatment are to be obtained only by the use of both human and bovine preparations alternately, and that certain cases should be treated first of all with *human* tuberculin. So I am not the blind advocate of bovine tuberculin your reviewer makes me out to be.

Next, he makes the following statement: "Is the balance of opinion among them in favour of I.K.? No, it is not." His assertion is absolutely incorrect. *The balance of medical opinion is decidedly in favour of I.K.*, and it is impossible for your reviewer or anyone else to escape from this fact, which can be proved by a scientific examination of the articles published by those who have used the treatment. Instead of making such an examination, which is laborious, your reviewer prefers the easier course of jumping to conclusions—a course which is neither scientific nor trustworthy. In my book I have shown that 65 per cent. of the reports are favourable. In the next edition this percentage will be higher, since during the past three years the percentage of favourable reports for each year has tended to become increasingly higher. Thus for the year 1912, 87.5 per cent. of those authors who have not previously written on the subject are favourable to I.K. In short, the percentage of favourable reports increases with the growth of scientific knowledge concerning the treatment.

In his attempt to criticise my figures, your reviewer charges me with the crime of having omitted five papers. Two of these (Baer, Starckloff) came out too late for me to obtain them for my book. Two of the three others appeared in Russian papers. Although I have had much help from friends on the Continent, your reviewer must know how exceedingly difficult—if not impossible—it is to obtain every paper on a subject within a year or two of its publication, especially those papers which are relatively unimportant and which are published in the less well-known medical journals. The *Centralblatt* on tuberculosis, which he quotes against me, has omitted at least fifteen papers.

Finally, your reviewer hints that my book has been written to induce those who are afflicted with tuberculosis to go to Switzerland. Since one of its main objects (see Chapter XVII.) is to show that with the adoption of more systematic and more scientific methods of early diagnosis the general practitioner by means of the immune substances (I.K.) treatment will be able successfully to treat his tuberculosis cases at home, I must ask your reviewer to revise his suppositions.—I am, etc.,

Frating, March 3. Walter H. Fearis.

* * * Mr. Fearis has written us a long letter, but we can answer him briefly.

1. As a layman (unless of the calibre of Metchnikoff) he must needs be lacking in acquaintance with modern medical research.

2. If he had more of that, he would know how common is the phenomenon of dominance by an *Orientirung*. Physiology says high altitudes produce erythrocythæmia. High altitudes are resorts for consumptives. Dr. Carl Spengler lives at high altitudes. He says that the red blood corpuscles are Nature's tuberculosis immunity producing factors. It may be true; we never said it was not—alluded to it, in fact, as work on its trial; but we assure Mr. Fearis that all through the history of medical research one can see the bleaching bones of many and many such a compact little theory.

3. We assure him also that many more have followed Bandelier and Rolpke, Penzoldt, Beraneck, Saph, and Morland than have followed Spengler, Wilkinson, and Raw.

4. We fear we prefer the selection of articles made by the leading record of tuberculosis literature to that made by Mr. Fearis.

But Mr. Fearis has omitted his best point, which is this: If a bad book, why review it at such length? In the first place, it is not a bad book; on the contrary, a carefully written one. But when a certain lay journal of standing took him so seriously as a populariser of important truths, it was felt that an example of the common folly of teaching professional people their own business badly needed making. This, and no absurd confusion of an able botanist with an hotel tout, made the reviewer write as he did.—The Reviewer.

